# Expression of IL-37 contributes to the immunosuppressive property of human CD4^+^CD25^+^ regulatory T cells

**DOI:** 10.1038/srep14478

**Published:** 2015-09-28

**Authors:** Xu Shuai, Li Wei-min, Ya-lin Tong, Ning Dong, Zhi-yong Sheng, Yong-ming Yao

**Affiliations:** 1 Trauma Research Center, First Hospital Affiliated to the Chinese PLA General Hospital, Beijing 100048, China; 2 Department of Hepatobiliary Surgery, the 309th Hospital of Chinese PLA, Beijing 100091, China; 3 Department of Burns and Plastic Surgery, the 181st Hospital of Chinese PLA, Guilin 541002, China

## Abstract

Interleukin-37 (IL-37) possesses the function of down-regulate systemic and local inflammation. It is unknown whether IL-37 is expressed in human regulatory T cells (Tregs) and its role in modulating the immune response of Tregs. In the present study, cell surface molecules and secretory cytokines were analyzed in order to determine the function of IL-37 in regulating inhibitory effect of human CD4^+^CD25^+^Tregs. Meanwhile, the effects of IL-37 on T cell differentiation and proliferation as co-culture of CD4^+^CD25^+^Treg/CD4^+^CD25^−^T cell were also investigated. It was showed that IL-37 was expressed in cytoplasm of CD4^+^CD25^+^Tregs, and the levels of IL-37 were gradually elevated with the enhanced activity of CD4^+^CD25^+^Tregs. Secretory cytokines such as transforming growth factor (TGF)-β and interleukin (IL)-10, and expressions of cell surface molecules, including forkhead/winged helix transcription factor p3 (FOXP3) and cytotoxic T-lymphocyte associated antigen (CTLA)-4, were significantly decreased when IL-37 gene was silenced by siRNA. Furthermore, down-regulation of IL-37 expression in human CD4^+^CD25^+^Tregs obviously promoted proliferation of co-cultured T cell and differentiation, together with observably enhancement of IL-2 formation. These results demonstrated that IL-37 might manifest as a critical protein involving in immunosuppression of human CD4^+^CD25^+^Tregs.

It is now well accepted that regulatory T cells (Tregs) are crucial to the proper maintenance of immune self-tolerance and homeostasis^1^,^2^. By drifting of helper T cell (Th)1/Th2 as a result of stimulation of T cell receptor signal, Tregs regulate immune system with immunosuppression^3^,^4^. Not only showing marked influence on immunosuppression, Tregs also play a role in developing immune nonreactivity. The phenomenon manifests as nonreactivity to antigenic stimulation and an interference of interleukin (IL)-2 expression, even stimulated by high concentration of IL-2. Tregs should be stimulated and proliferated, however, the level of proliferation is obviously lower than that of CD4^+^CD25^−^T cells.

Tregs are different from other regulatory or suppressor cells by possessing particular immunological characteristics. Tregs can express different kinds of cell molecules. Some molecules promote the growth and liveness of cells, including Toll-like receptors (TLRs) as well as forkhead/winged helix transcription factor p3 (FOXP3), which identify pathogen associated molecule patterns or promote Treg function or proliferation. Tregs also persistently express some other factors including glucocorticoid-induced tumor necrosis factor (TNF) receptor (GITR) and intracellular cytotoxic T-lymphocyte-associated antigen (CTLA)-4. In addition, Tregs can produce various immunosuppressive cytokines, including transforming growth factor (TGF)-β and IL-10, and they might also contribute to the inhibition of effector T cells^5^,^6^,^7^,^8^,^9^,^10^.

IL-37, which is the seventh interleukin factor of interleukin 1 family (IL-1F7), has the ability to down-regulate systemic and local inflammation by lowering levels of pro-inflammatory mediators^11^,^12^. Also, it is involved in both innate and adaptive immunity. With evidences accumulated, IL-37 is recognized as a typical anti-inflammatory cytokine related to the autoimmune disease, endotoxemia, liver inflammatory injury, obesity, and cancer^13^,^14^,^15^,^16^,^17^,^18^.

In the past decade, many investigators demonstrated the anti-inflammatory property of IL-37. IL-37 suppresses the production of various pro-inflammatory cytokines, including IL-1α, IL-1β, IL-6, IL-12, granulocyte colony-stimulating factor (G-SCF), granulocyte-macrophage colony-stimulating factor (GM-CSF), and TNF-α. However, this property does not depend on the production of anti-inflammatory cytokines, such as IL-10^12^. IL-37 also inhibits activation of dendritic cells (DCs), thus playing a role in adaptive immunity^12^,^19^. It has been documented that IL-37 forms an intracellular functional complex with Smad-3, relevant gene transcription is affected. Extracellular IL-37 binds to IL-18-binding protein (IL-18BP), and subsequently binds IL-18Rb, resulting in the inhibition of the pro-inflammatory activity of IL-18. In addition, IL-37 binds to the IL-18R a-chain, but with much lower affinity than that of IL-18^12^,^20^,^21^,^22^.

Because expression of IL-37 in the immune system keeps immune homeostasis, we supposed that IL-37 might be involved in the immune regulation processed by Tregs. The objective of this study was to identify IL-37expression in human CD4^+^CD25^+^Tregs with Western blotting and confocal laser scanning microscopy, and further investigate the potential effect of IL-37 on Treg-mediated immunosuppression *in vitro*.

## Results

### Expression of IL-37 in human CD4^+^CD25^+^Tregs

**Figure 1:**
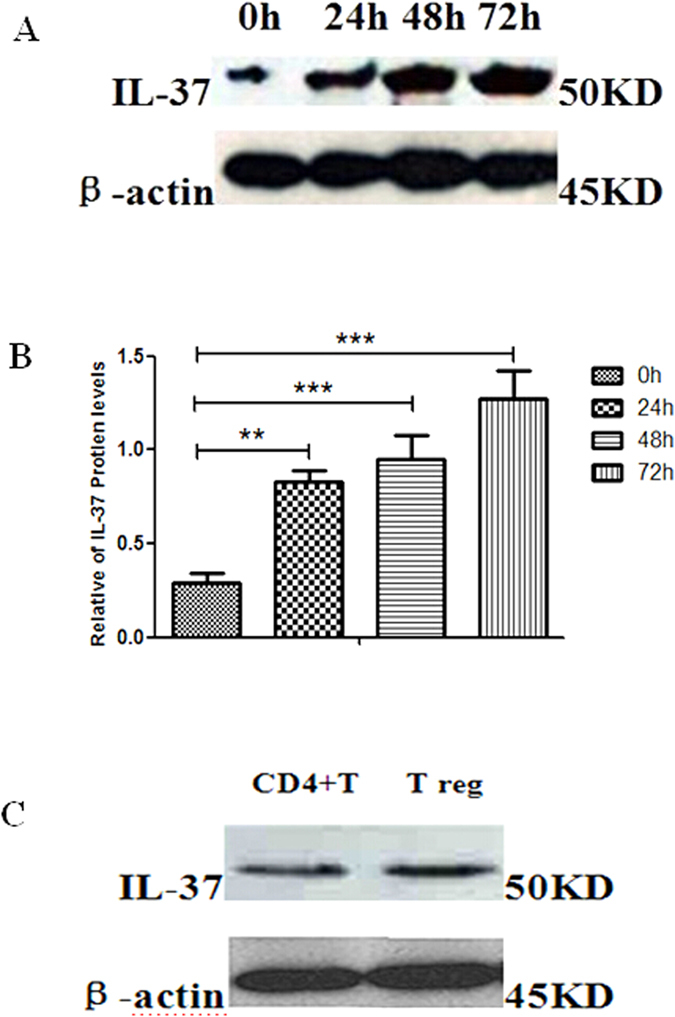
The expression levels of IL-37 in humanCD4^+^T cells and CD4^+^CD25^+^Tregs were showed in Fig. 1. (**A**) Western blotting gel revealed that in fresh extraction of CD4^+^CD25^+^Tregs group (0 h) IL-37 showed a small amount of expression. Under the sustaining stimulation of human Treg expander, Western blotting gel showed that the protein levels of IL-37 in CD4^+^CD25^+^Tregs were gradually increased. (**B**) Relative abundance of IL-37 protein levels in CD4^+^CD25^+^Tregs. Representative Western blotting gel showed relative abundance of IL-37 protein levels in CD4^+^CD25^+^Tregs under the sustaining stimulation of human Treg expander for 24 h, 48 h, and 72 h, respectively. Compared with the 0 h controls, **P < 0.01; ***P < 0.001. (**C**) Western blotting gel revealed that CD4^+^T cells and Tregs which were respectively cultured with a combination of soluble anti-CD3/anti-CD28 monoclonal antibodies and Treg expander for 24 h contained a clear protein level of IL-37.

**Figure 2:**
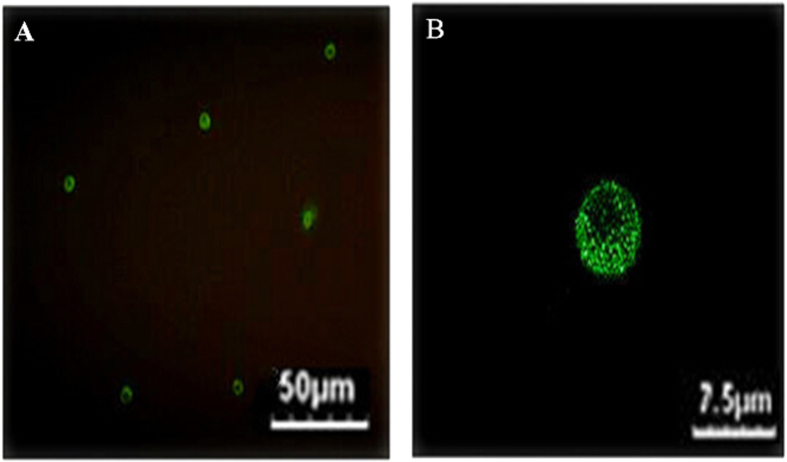
IL-37 expression in CD4^+^CD25^+^Tregs as shown by confocal laser scanning microscopy. After stimulation with human Treg expander for 72 h, IL-37 protein expression in CD4^+^CD25^+^Tregs was determined with confocal laser scanning microscopy. IL-37 protein was shown by antibodies indirectly labelled with FITC (green) (**A**,**B**). Representative photomicrographs showed that FITC-positive cells (green) were detected among CD4^+^CD25^+^Tregs. The cells in (**A**) were magnified in (**B**). As shown in (**B**) it was found that IL-37 was a cytoplasmic protein and expressed abundantly in stimulated CD4^+^CD25^+^Tregs.

The expression of IL-37 in humanCD4^+^CD25^−^T cells and CD4^+^CD25^+^Tregs was assessed by dint of Western blotting through the specific IL-37 antibody (Ab). As shown in Fig. 1, in the fresh extraction of CD4^+^CD25^+^Tregs group (0 h), a distinct band with a molecular mass of approximately 45 kDa from CD4^+^CD25^+^Tregs was found. Under the sustaining stimulation of human Treg expander, it was showed that the protein expression levels of IL-37 in CD4^+^CD25^+^Tregs were gradually increased. CD4^+^T cells and Tregs which were respectively cultured with a combination of soluble anti-CD3/anti-CD28 monoclonal antibodies and Treg expander for 24 h, contained a clear protein levels of IL-37. Protein level of β-actin was regarded as an internal control. Meanwhile, positive expression of IL-37 was found with confocal laser scanning microscopy. In Fig. 2, after 72 h sustaining stimulation of human Treg expander, green fluorescence was observed in the cytoplasm of the 72 h group of CD4^+^CD25^+^Tregs. These findings proved that IL-37 was poorly expressed in inactive CD4^+^CD25^+^Tregs, nevertheless, with gradually increased activity of CD4^+^CD25^+^Tregs, IL-37 was markedly expressed.

### The effects of IL-37 on T-cell proliferation and differentiation mediated by CD4^+^CD25^+^Tregs

**Figure 3:**
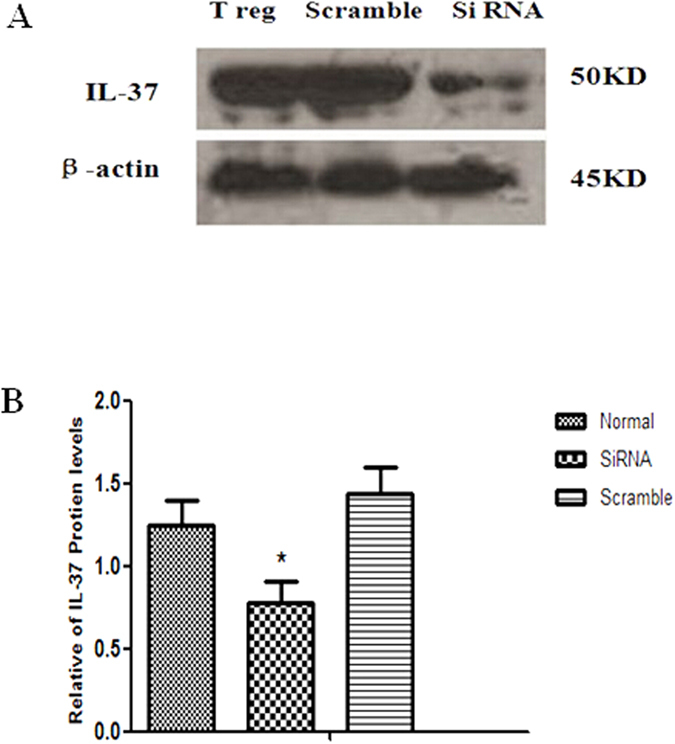
Relative abundance of IL-37 protein levels in CD4^+^CD25^+^Tregs. Representative Western blotting gel showed relative abundance of IL-37 protein levels in CD4^+^CD25^+^Tregs (normal), siRNA-IL-37, and Scrambled control RNA transfected CD4^+^CD25^+^Tregs. Compared with the normal controls, *P < 0.05.

**Figure 4:**
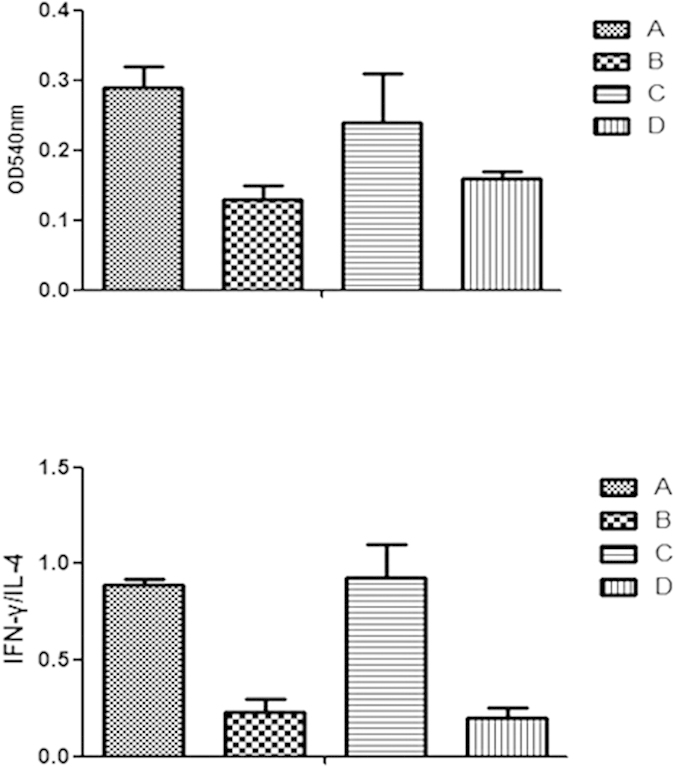
Effects of IL-37 on the T-cell proliferation and differentiation mediated by CD4^+^CD25^+^Tregs. CD4^+^CD25^−^T cells were activated with a combination of soluble anti-CD3 and anti-CD28 monoclonal antibodies (**A**). CD4^+^CD25^+^Tregs were mixed with CD4^+^CD25^−^T cells at a ratio of 1:1 in the presence of anti-CD3/CD28 antibodies (**B**). siRNA-IL-37 transfected CD4^+^CD25^+^Tregs stimulated with anti-CD3/CD28 antibodies were co-cultured with CD4^+^CD25^−^T cells (**C**). Scrambled control RNA transfected CD4^+^CD25^+^Tregs stimulated with anti-CD3/CD28 antibodies were co-cultured with CD4^+^CD25^−^T cells (**D**). A modified 3-(4,5-dimethylthiazol^−^2-yl) -2,5-diphenyltetrazolium bromide (MTT) assay was used to measure T-cell proliferation activity. Levels of IFN-γ and IL-4 in culture medium were determined by ELISA, and IFN-γ/IL-4 ratio was used to evaluate T-cell differentiation. Results of independent experiments were shown as the mean ± SD. Statistically significant difference was found between the values in groups (**B,C**) (P < 0.05).

It is well known that Tregs are dull to mitogenic stimuli and inhibitors to T effect cell proliferation. Because CD4^+^CD25^+^Tregs could express IL-37, we further investigated whether IL-37 might be related to the regulation of Treg suppressive character. In this part of experiment, IL-37 gene was silenced by siRNA. Western blot analysis showed that IL-37 expression was markedly down regulated compared with the normal control in siRNA-IL-37 transfected CD4^+^CD25^+^Tregs (Fig. 3, P < 0.05). Under stimulation of anti-CD3/CD28 Abs, co-culture of CD4^+^CD25^+^Tregs and CD4^+^CD25^−^T cells (1:1 ratio) showed a decreased proliferation capacity of CD4^+^CD25^−^T cells. By comparison, a significantly increased proliferation of CD4^+^CD25^−^T cells was noted when expression of IL-37 was silenced by siRNA in Tregs (P < 0.05, Fig. 4). What were showed above indicated that IL-37 might be involved in the function of Tregs to suppress T cells, thus promoting the immune suppressive capacity of Tregs.

We know that Th1 cells secrete IFN-γ, and Th2 cells secrete IL-4. Thus, a ratio of IFN-γ/IL-4 is able to identify polarization of naive T cells. In the present study, ELISA was used to measure the two cytokines. When co-cultured with CD4^+^CD25^−^T cells, CD4^+^CD25^+^Tregs up-regulated IL-4 level and down-regulated IFN-γ level significantly (Fig. 4). Besides, it showed in Fig. 4B, a remarkable rise in IFN-γ/IL-4 ratio when expression of IL-37 was inhibited by siRNA in Tregs (P < 0.05). The results suggested that IL-37 might be involved in the process of CD4^+^CD25^+^Tregs induced type 2 T-cell polarization.

### The effect of IL-37 on expression of CTLA-4 and FOXP3 in CD4^+^CD25^+^Tregs

**Figure 5:**
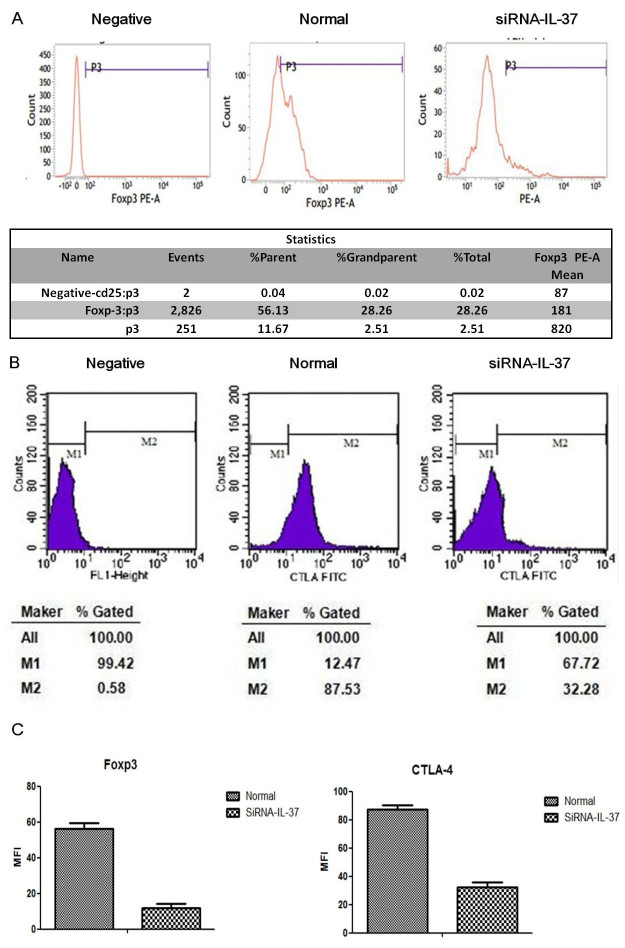
Effects of IL-37 on CTLA-4 and FOXP3 expression in CD4^+^CD25^+^Tregs. Normal condition means that after being isolated from human PBMCs, Tregs were cultured with Treg expander for 24 h; si-RNA-IL-37 condition means that silenced Tregs were cultured with Treg expander for 24 h. Expressions of CTLA-4 and FOXP3 were determined by flow cytometry (**A**,**B**). Results of independent experiments were shown as the mean ± SD. Statistically significant difference when compared with normal controls, P < 0.05 (**C**).

In order to explore the effect of IL-37 on cell molecules of Tregs, levels of CTLA-4 and FOXP3 were analyzed by flow cytometry. As shown in Fig. 5, CTLA-4 expression on CD4^+^CD25^+^Tregs was significantly down-regulated when gene of IL-37 was silenced. Similarly, gene silence of IL-37 lowered the expression of FOXP3 in CD4^+^CD25^+^Tregs.

### The effect of IL-37 on formation of TGF-β as well as IL-10 in CD4^+^CD25^+^Tregs

**Figure 6:**
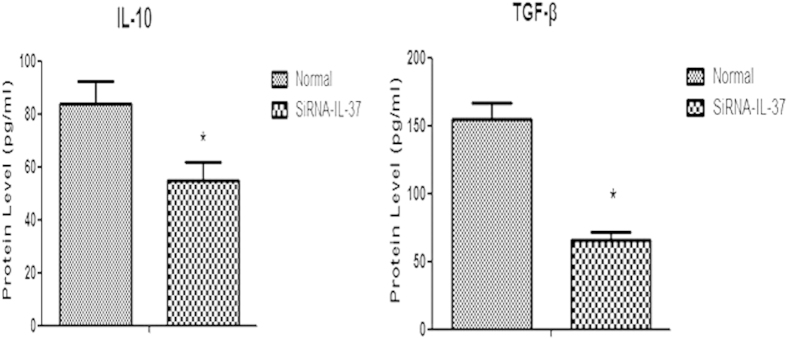
Effects of IL-37 on production of TGF-β and IL-10 in CD4^+^CD25^+^Tregs. Normal condition means that after being isolated from human PBMCs, Tregs were cultured with Treg expander for 24 h; si-RNA-IL-37 condition means that silenced Tregs were cultured with Treg expander for 24 h. The production of TGF-β and IL-10 was determined by ELISA. Results of independent experiments were shown as the mean ± SD. Statistically significant difference when compared with normal controls, *P < 0.05.

Under costimulation of anti-CD3/CD28 Ab, CD4^+^CD25^+^Tregs produce high levels of TGF-β as well as IL-10. However, as shown in Fig. 6, secretion of TGF-β and IL-10 in CD4^+^CD25^+^Tregs transfected by siRNA-IL-37 was prominently reduced compared to normal controls. These data indicated that IL-37 could affect the formation of anti-inflammatory cytokines in CD4^+^CD25^+^Tregs.

### The effect of IL-37 on IL-2 formation in CD4^+^CD25^**−**^T cells in co-culture experiments

**Figure 7:**
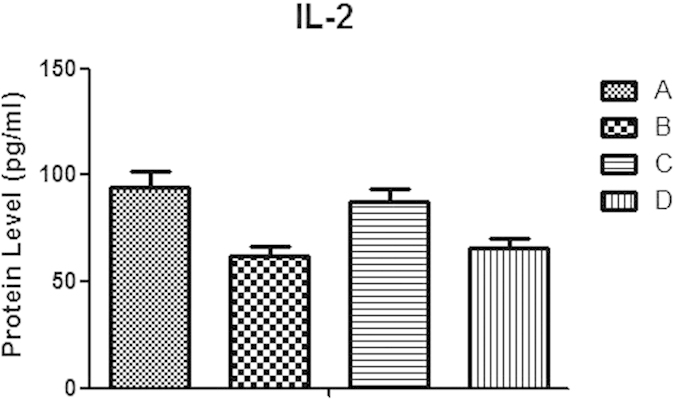
Effects of IL-37 on IL-2 formation in CD4^+^CD25^−^T cells in co-culture experiments. CD4^+^CD25^−^T cells were activated with a combination of soluble anti-CD3 and anti-CD28 monoclonal antibodies (**A**). CD4^+^CD25^+^Tregs were mixed with CD4^+^CD25^−^T cells at a ratio of 1:1 in the presence of anti-CD3/CD28 antibodies (**B**). siRNA-IL^−^37 transfected CD4^+^CD25^+^Tregs stimulated with anti-CD3/CD28 antibodies were co-cultured with CD4^+^CD25^−^T cells (**C**). Scrambled control RNA transfected CD4^+^CD25^+^Tregs stimulated with anti-CD3/CD28 antibodies were co-cultured with CD4^+^CD25^−^T cells (**D**). IL-2 levels in the conditioned media were measured by ELISA. Results of independent experiments were shown as the mean ± SD. Statistically difference between the values in groups (**B,C**) (P < 0.05).

As a crucial T-cell growth factor, IL-2 acts upon itself in an autocrine modality. CD4^+^CD25^+^Tregs were co-cultured with CD4^+^CD25^−^T cells under stimulation of anti-CD3/CD28 Abs. 72 h later, supernatants were collected for measurement of IL-2 released from CD4^+^CD25^−^T cells. The formation of IL-2 was determined after CD4^+^CD25^−^T cells co-cultured with IL-37 silenced CD4^+^CD25^+^Tregs. It was shown that IL-2 levels in CD4^+^CD25^−^T cells were lowered when co-cultured with CD4^+^CD25^+^Tregs (Fig. 7). However, IL-2 level in T cells was prominently elevated (P < 0.05) when IL-37expression was suppressed by siRNA in Tregs.

## Discussion

IL-37 is the 7th factor (IL-1F7) of interleukin-1 family (IL-1F), and it is widely expressed in human organs and tissues. However, it does not exist in mouse for the lack of its locus^23^. Since the first report of IL-37 in 2000^24^, there has been many studies with regard to the anti-inflammatory effect of endogenous IL-37. Cysteinyl aspartate-specific protease (caspase)-1 processing is required for maturation of the intracellular IL-37 precursor and for the translocation of the cytokine to the nucleus^25^. *In vitro*, down-regulation of IL-37 in human peripheral blood mononuclear cells (PBMCs) increased the synthesis of various pro-inflammatory mediators, and it does not depend on the formation of anti-inflammatory cytokines such as IL-10. *In vivo*, injection of inflammatory cytokines towards transgenic for IL-37tg and control mice, the pro-inflammatory effect of the stimuli was markedly suppressed by IL-37b^12^. IL-37 expression in DCs not only promoted generation of semi-mature tolerogenic DCs, and it also suppressed antigen-specific immune response^19^. It has been demonstrated that Smad-3, a transcriptional modulator in TGF-β pathway, plays a significant role in the biological effects of IL-37b by contributing translocation of IL-37b to the nucleus^26^. A growing body of evidence indicates that IL-37 appears to be related to the autoimmune disease, endotoxemia, and liver inflammatory injury^13^,^14^,^15^,^16^,^17^,^18^. It was revealed that IL-37 expression in the tumor microenvironment might inhibit liver tumor growth by recruiting NK cells into tumor tissues^27^. More recently, Nold *et al.* and Li *et al.* had demonstrated that IL-37 might act as an extracellular cytokine by binding receptors IL-18α and IL-1R8 (SIGIRR) for a multifaceted intracellular anti-inflammatory process^28^,^29^, which was in accordance with our viewpoint in the current study. However, the potential significances and underlying mechanisms of IL-37 in mediating immune suppressive function of Tregs are poorly known.

CD4^+^CD25^+^Tregs have been confirmed to perform main functions in processes of immune tolerance and regulation, including infection, inflammation/injury, tumor persistence/progression, and transplant tolerance^30^,^31^,^32^. Firstly, in the current study, it was noted that IL-37 maintained a weak expression in the fresh extraction of human CD4^+^CD25^+^Tregs without stimulation. With the sustaining stimulation of human Treg expander, the expression of IL-37 in Tregs was distinctly increased. We found that CD4^+^T cells which cultured with a combination of soluble anti-CD3/anti-CD28 monoclonal antibodies for 24 h, also contained a clear protein levels of IL-37. The functions of IL-37 in CD4^+^T cell are being studied by us. To further confirm the location and level of IL-37 in Tregs, IL-37 protein were determined by confocal laser scanning, and it showed that positive green fluorescence existed in the cytoplasm of Tregs under Treg expander stimulation for 72 h. Moreover, IL-37 was markedly expressed along with enhanced activity of CD4^+^CD25^+^Tregs. As far as we know, this is the first report to state IL-37 could be definitely expressed in human CD4^+^CD25^+^Tregs. Thus, IL-37 appears to be an important regulator related to immunodepression of CD4^+^CD25^+^Tregs.

Nonreactivity to the stimulation of IL-2 are the unique immunologic characteristics of CD4^+^CD25^+^Tregs in contrast to other immune cells. Also, Tregs mainly regulate the drifting of CD4^+^T cell differentiation as well as immunosuppression. It is well known that IL-37 is substantially expressed in human PBMCs and DCs, and it plays anti-inflammatory effects in maintaining immune homeostasis^12^,^19^. Based on our own studies, demonstrating the presence of positive IL-37 expression in human CD4^+^CD25^+^Tregs, we speculated that IL-37 might play a regulatory role in immunosuppression of CD4^+^CD25^+^Tregs.

It has been well known that CD4^+^CD25^+^Tregs exert immune suppressive activity through various mechanisms. As a particularly specific marker, the nuclear transcription factor FOXP3 is closely related to the development and function of Tregs. Mutation in FOXP3 gene could result in a maturity defection of Tregs and the performance of a deadly autoimmune disorder^6^,^33^. We noticed that down-regulation of IL-37 markedly reduced FOXP3 level in CD4^+^CD25^+^Tregs, indicating that IL-37 might be an crucial factor in activating immunosuppression of CD4^+^CD25^+^Tregs. Similar to FOXP3, Tregs are enriched of CTLA-4 expression, which might be associated with lymphopenia-induced proliferation of CD4^+^T cells^34^ The results of present study showed that a down regulated expression of IL-37 could prominently lower CTLA-4 level on CD4^+^CD25^+^Tregs. At the same time, the immunosuppression mediated by CD4^+^CD25^+^Tregs was obviously diminished. Therefore, changes in CTLA-4 as well as FOXP3 expressions imply that IL-37 participates in immune response of CD4^+^CD25^+^Tregs, in turn resulting in immunosuppression.

IL-10 and TGF-β are immune suppressive cytokines which are critically related to playing the tolerance speciality of Tregs^35^. In the present study, both IL-10 and TGF-β secreted levels, which measured in supernatants of CD4^+^CD25^+^Tregs as IL-37 inhibited, were significantly reduced as to their release under normal condition. These findings indicated that the lowered expression of IL-37 in CD4^+^CD25^+^Tregs might be involved in reduction of anti-inflammatory cytokines.

Furthermore, Tregs also prominently induced an immune homeostasis, including nonreactivity to the antigenic stimulation as well as the control of IL-2 formation and consumption of excessive IL-2^34^,^35^,^36^. Because IL-2 generates indispensable stimulations for Tregs developed from thymus, we would like to know whether IL-37 could influence the production of IL-2. Our data showed that secreted levels of IL-2 in siRNA-IL-37 transfected CD4^+^CD25^+^Treg/CD4^+^CD25^−^T cell co-culture group were prominently rose, documenting that down-regulation of IL-37 level appears to be involved in the increase in IL-2 secretion, thereby resulting in a weaker CD4^+^CD25^+^Treg-mediated immune depression towards CD4^+^T cells.

Co-culture of CD4^+^CD25^+^Tregs and CD4^+^CD25^−^T cells showed a decreased proliferative capacity of CD4^+^CD25^−^T cells. By contrast, a significantly increased proliferation of CD4^+^CD25^−^T cells was found when IL-37 was silenced in CD4^+^CD25^+^Tregs. It indicated that IL-37 might be involved in the function of Tregs to suppress T cells, thus promoting the immune suppressive capacity of Tregs. Nonetheless, there are method limitations in the present study that the MTT assay can read the proliferative activity both Tregs and conventional T cells, it is possible that Tregs might proliferate more when FOXP3 were down-regulated.

Stimulated by different kinds of cytokines as well as co-stimulating molecules, CD4^+^T cells can differentiate into groups of T cells, in terms of the levels of IFN-γ by Th1, and IL-4 as well as IL-10 by Th2^37^,^38^,^39^,^40^. Activated Tregs can mediate immunosuppressive effect by shifting the ratio of Th1/Th2, which leads to the signal activation of different T cell receptors. Herein we explored whether IL-37 expressed in CD4^+^CD25^+^Tregs had an impact on mediating drift of Th1/Th2. In the current experiment, the supernatants from co-culture cells were collected to analyze the quantities of cytokines generated from Th1 and Th2. It was interesting to notice that co-culture of CD4^+^CD25^−^T cells and siRNA-IL-37 transfected CD4^+^CD25^+^Tregs for three days performed an increase toward Th1 cytokine pattern in T-cell polarization, as indicated by rise of IFN-γ/IL-4 ratios. On the basis of the results, we believe that IL-37 might play a significant role in the cell drifting toward Th2 in the CD4^+^CD25^+^Treg-mediated immune suppressive process.

In conclusion, the present study *in vitro* demonstrates that IL-37 expression in human CD4^+^CD25^+^Treg can promote the suppressive effect on T lymphocyte activation. An understanding of the key role of IL-37 in the immunosuppressive activity of human CD4^+^CD25^+^Tregs may expand our view for further research regarding immune homeostasis and immune related diseases. Nevertheless, more investigation is required to study the precise mechanism underlying IL-37 on CD4^+^CD25^+^Tregs including intracellular as well as extracellular functions and the potential effect associated with host immune response *in vivo*.

## Materials and Methods

This study was conducted according to the principles expressed in the Declaration of Helsinki. The manuscript statements of informed consent were obtained from all blood donors. This study was approved by the Ethics Committee and Scientific Investigation Board of the Chinese People’s Liberation Army 309th Hospital, Beijing, China.

### Blood samples

15 ml of peripheral venous blood was obtained from each of 21 healthy donors (16 males, 5 females), who were proved healthy in the Chinese People’s Liberation Army 309th Hospital in Beijing, China.

### Cell isolation and purification

Blood sample was collected in 15 ml tubes with EDTA. PBMCs were isolated from peripheral venous blood by density gradient centrifugation, and then CD4^+^CD25^+^Tregs were purified.

Human CD4^+^CD25^+^Tregs were isolated by CD4^+^CD25^+^Tregs isolation kit (MiltenyiBiotec GmbH, Bergisch Gladbach, Germany). PBMCs were stained by biotin-antibody cocktail (10 μl/10^7^ total cells) and then incubated for 10 min at 2–8 °C in the dark. They were then incubated with anti-biotin microbeads (20 μl/10^7^ total cells) for 10 min at 2–8 °C in the dark. The prepared cells flowed through a magnetic cell sorting (MACS) column (the purity of human CD4^+^T cells >90%). The collected CD4^+^CD25^−^T cells were labelled with CD25 microbeads (10 μl/10^7^ total cells) and further isolated by MACS. Flow cytometry determined the purity of CD4^+^CD25^+^Tregs (>93%). Trypan blue was used to identify the cellular activity.

### Immunoblotting and confocal microscopy analysis

Immunoblotting and confocal microscopy analysis were used to analysis the expression of IL-37 in human CD4^+^CD25^+^Tregs. Tregs were resuspended in lysis buffer which contained protease inhibitor mixture. Then cells were centrifuged at 10,000 × g for 15 min at 4 °C. samples were added on 10% SDS gels, and then, gels were electro-transferred to an immobilonpolyvinylidene difluoride membrane. Blocking with milk overnight on ice, the membrane was incubated with anti-37 polyclonal Ab (Abcam, Cambridge, UK) (1:500) for 4 h at room temperature or anti-β-actin as control group. Membrane incubated with peroxidase-labelled affinity secondary Ab for 1 h after washed three times. Then, the membrane was processed by a ECL plus chemiluminescence kit (Amersham Biosciences, Uppsala, Sweden). In the process of confocal microscopy analysis, CD4^+^CD25^+^Tregs were activated for 48 h, and then, cells were fixed with 4% paraformaldehyde in PBS for 20 min. After being permeabilized with 0.02% Triton X-100 for 20 min at room temperature, the cells were blocked with bovine serum albumin for 30 min and incubated with anti-IL-37 polyclonal Ab (Abcam, Cambridge, UK) (1:200) overnight at 4 °C. After being stained with goat FITC-anti-IgG as the second Ab for 1 h at room temperature, samples were observed with a laser scanning confocal microscope (Leica, Mannheim, Germany).

### RNA interference

Small interference RNA (siRNA) of IL-37 was obtained from Thermo Fisher Scientific Co.^12^. This kind of 100 nM siIL-37 targeted all isoforms of IL-37, comprised 25 nM of the four following antisense sequences: I, 5′-UCAAGGAUGAGGCUAAUGCUU-3′; II, 5′-CAAUGUGUUUCCUGUUCUCUU-3′; III, 5′-UUACAAUUGCAGGAGGUGCUU-3′; IV, 5′-UUAUCCUUGUCACAGUAGAUU-3′. Electroporation was performed by Amaxa device. After overnight recovery, CD4^+^CD25^+^Tregs were co-cultured with siIL-37 for 72 h to knock-down IL-37 expression. Concomitantly, the same number of control siRNA as wild type control was transfected. The efficiency of knock-down was measured by immunoblotting. In this part experiment, we found that the Treg cells survival rate declined obviously. And about 30% cells dead after being silenced by IL-37-siRNA or scramble-siRNA. So we removed the dead cells and collected living cells for next experiment. Trypan blue was used to identify the cellular activity.

### Cell stimulation and suppression assay *in vitro*

Human CD4^+^CD25^−^T cells and CD4^+^CD25^+^Tregs were co-cultured in 96-well culture plate (1:1 ratio). These cells were stimulated with a combination of 1 μg/ml of soluble anti-CD3 and anti-CD28 monoclonal Abs (BD Biosciences, Mountain View, CA) at 37 °C and 5% CO_2_ for 72 h. Thereafter, modified3-(4,5-dimethylthiazol-2-yl)-2,5-diphenyltetrazolium bromide (MTT) (5 mg/ml, 10 μl/well)^23^,^41^,^42^,^43^ was added in each sample and the incubation continued for 4 h. The suspending crystals were blown by pipette repeatedly, then optical density of samples were separately measured by microplate reader under the wave length of 540 nm. These experiments were repeated three times and results were the mean of three samples.

### Flow cytometric analysis

Fluorescein isothiocyanate (FITC)-CTLA-4 Ab as well as PE-FOXP3 Abs (BD Pharmingen, San Diego, CA) were used for flow cytometric analysis. Stimulated CD4^+^CD25^+^Tregs (10^6^) were stained by FITC-CTLA-4 Ab or FITC-conjugated anti-Armenian hamster Ab as an isotype control. Analytical procedure of FOXP3 was different. After disposing with fresh prepared fixation/permeabilization working solution, samples were washed with permeabilization buffer and incubated with PE-FOXP3 Ab at 4 °C for 30 min in the dark. At last, after washing cells, samples were fixed in 1% formaldehyde solution and analyzed by FACSCalibur (BD Biosciences, Mountain View, CA).

### Cytokine measurement by ELISA

To quantify the secreted cytokines, cocultured cells (1:1 ratio) of human CD4^+^CD25^−^T cell and CD4^+^CD25^+^Treg were stimulated by anti-CD3 and anti-CD28 monoclonal Abs (1 μg/ml) in 96-well plate for 68 h at 37 °C and 5% CO_2_ in triplicate. 100 μl supernatant was collected from each sample. Supernatant for measurement of IL-10 and TGF-β were collected at 24 h in group of CD4^+^CD25^+^Tregs. Secretion of IL-2, IL-4, IFN-γ, IL-10 and TGF-β were determined by ELISA kits (R&D Systems, Minneapolis, MN). Microplate reader (Spectra MR, Dynex, Richfield, MN) showed the results.

### Statistics analysis

Quantitative values, with the respective experiment repeated triplicate, were expressed as the mean ± standard deviation (SD). A paired two-tailed Student t test was performed in cases. The differences between multiple groups were analyzed by one-way ANOVA. The p-value less than or equal to 0.05 was considered significant.

## Additional Information

**How to cite this article**: Shuai, X. *et al.* Expression of IL-37 contributes to the immunosuppressive property of human CD4^+^CD25^+^ regulatory T cells. *Sci. Rep.* **5**, 14478; doi: 10.1038/srep14478 (2015).
